# Safety of a novel feed ingredient, Algal Oil containing EPA and DHA, in a gestation-lactation-growth feeding study in Beagle dogs

**DOI:** 10.1371/journal.pone.0217794

**Published:** 2019-06-03

**Authors:** Irina Dahms, Eileen Bailey-Hall, Erin Sylvester, Audrey Parenteau, Shiguang Yu, Alexios Karagiannis, Franz Roos, Jon Wilson

**Affiliations:** 1 DSM Nutritional Products, Kaiseraugst, Switzerland; 2 DSM Nutritional Products, Columbia, Maryland, United States of America; 3 Citoxlab North America, Laval, Quebec, Canada; University of Illinois, UNITED STATES

## Abstract

An Algal Oil Containing EPA and DHA (AOCED) at ~50% was developed as a sustainable source of omega-3 fatty acids. AOCED was incorporated into extruded dry foods for dogs at 0, 0.75%, 1.5% and 3.0% levels (equivalent to 0, 7.5, 15 and 30 g/kg diet) on dry matter basis at the expense of chicken fat and fed to healthy female Beagle dogs starting at mating and throughout gestation and lactation. The offspring were fed their maternal corresponding diets for 26 weeks after weaning. AOCED-enriched diets were well tolerated by dogs in both generations and did not affect their overall health, physiological parameters, food consumption, body weights and body weight gains. There were no changes in hematology, clinical chemistry, and coagulation parameters in both generations of dogs fed the AOCED diets when compared to those in the control group. Plasma levels of DHA and EPA increased significantly and generally dose-dependently in both generations. The study demonstrated the safety of AOCED in dogs during gestation, lactation, and growth periods at dietary levels up to 3.0wt%, equivalent to 30 g/kg diet. AOCED’s bioavailability as a source of DHA and EPA in dogs was demonstrated by the increased plasma concentrations of these nutritional lipids.

## Introduction

There is ongoing interest in the role of long-chain polyunsaturated n-3 fatty acids (n-3 LC-PUFA) docosahexaenoic acid (DHA, 22:6n-3) and eicosapentaenoic acid (EPA, 20:5n-3) in companion animal nutrition. Evidence has accumulated regarding DHA and EPA having a range of physiological roles that relate to optimal cell membrane structure and beneficial cell function and responses. DHA plays an essential role in the development of the nervous system [1], and along with EPA it is thought to modulate immune functions and reduce inflammatory responses [2].

Benefits of dietary supplementation with n-3 LC-PUFA have been documented in several species, including dogs and cats (reviewed in [3]). DHA and EPA are recommended for dogs and cats in the Nutrient Requirements of Dogs and Cats published by the National Research Council (NRC) [4]. Based on the scientific evidence indicating that n-3 LC-PUFAs are needed for the development of the nervous and visual systems during fetal and neonatal life stages, the Canine Nutrition Expert Subcommittee of the Association of American Feed Control Officials (AAFCO) established a dietary minimum concentration of EPA plus DHA at 0.05% on dry matter (DM) basis for growing and reproductive dogs [5]. Most canine studies have been conducted with fish oils as omega-3 PUFA sources, and in some of them relatively high doses of omega-3 fatty acids were used for the management of several conditions, such as joint health, osteoarthritis, atopic dermatitis and cardiovascular, renal, and metabolic disorders [6–10]. High doses of fish oil are not without risks and their potential adverse effects in companion animals were reviewed initially by Hall [11] and later by Lenox and Bauer [12]. Among detrimental effects which may occur with high dose supplementation of LC-PUFA, changes in coagulation and immune functions were suggested, as well as gastrointestinal disturbances and some other effects resulting from rancidity of LC-PUFA [12]. NRC set a dietary safe upper limit of EPA plus DHA for adult dogs at 11g/kg on a DM basis or 1.1% DM [4].

Although most commercial pet foods contain a source of n-3 LC-PUFA, this source is primarily fish oil obtained from fatty fish, such as menhaden, anchovies, herring and mackerel. High demand for fish meal and oil for both human and animal nutrition increases the impact on ocean resources, endangering fish stocks [13]. However, fish do not synthesize these fatty acids *de novo*, lacking the required key enzymatic activities [14]. Instead, fish obtain DHA and EPA from their diet in the form of plankton/single cell algae. Large-scale fermentation technology has made it possible to by-pass the marine food chain and produce DHA and EPA oils directly from microalgae. An Algal Oil Containing EPA and DHA (AOCED) produced from *Schizochytrium* sp. was developed as an alternative source of LC-PUFA without risk of environmental contaminants such as heavy metals and PCB’s. It enables the animal nutrition industry to keep up with the increasing demand for EPA and DHA without endangering fish stocks.

An algal oil similar to AOCED and also produced from the same species of *Schizochytrium* (named Algal Oil hereafter) has been used in human nutrition as a food ingredient and dietary supplement. Results of the comprehensive Algal Oil toxicity testing including a 90-day subchronic dietary study in rats and three genetic toxicity assays have been published [15]. Algal Oil was non-mutagenic with respect to gene mutations, clastogenicity and aneugenicity, confirming that algal oils do not have genotoxic potential. The results of the rat study demonstrated safety of Algal Oil at extremely high intake levels. There were no treatment-related effects noted at any dose level. Based on these results, the no observable adverse effect level for Algal Oil was established at 5 wt% in the diet, or at the highest tested dose. This dose corresponded to an overall average Algal Oil intake of 3250 mg/kg bw/day for male and female rats.

The objective of the present study was to evaluate safety and bioavailability of AOCED as a source of dietary EPA and DHA given to beagle dogs starting at mating, throughout gestation, lactation of dams and growth of their offspring. After weaning, puppies selected for the study continued on their mothers’ respective diets for additional 26 weeks. AOCED was incorporated onto extruded dry dog food at three dose levels, 0.75%, 1.5% and 3.0% of the diet on Dry Matter (DM) basis. Dose levels were selected based on data collected during the survey of labels of dog foods conducted by DSM in December 2016. The mid-dose (1.5%) was defined as the dose which provides the actual maximum dose of EPA and DHA currently available in non-therapeutic dog and cat diets marketed worldwide. The low- and high-doses were selected to provide information on dose-response relations of test item-related findings, if any.

## Materials and methods

This study was conducted at Citoxlab North America (Laval, Quebec, Canada) according to their standard operating procedures but not under good laboratory practice regulations. The study was designed with the reference to the following guidelines: CVM guidance for industry #56: protocol development guideline for clinical effectiveness and target animal safety trials; CVM guidance for industry #221: recommendations for preparation and submission of animal food additive petitions; VICH GL43 (CVM GFI #185): guidance for industry: target animal safety for veterinary pharmaceutical products; AAFCO dog food gestation/lactation and growth feeding protocols. The CVM is the Center of Veterinary Medicine, a division of the US Food and Drug Administration (FDA). Procedures involving the care and use of animals in the study were approved by the Institutional Animal Care and Use Committee prior to conduct. During the study, the care and use of animals were conducted in accordance with the principles outlined in the current guidelines published by the Canadian Council on Animal Care and the guide for the care and use of laboratory animals, an NRC publication and USDA regulations. Citoxlab North America facility is accredited by the Canadian Council on Animal Care and AAALAC.

### Animals and housing

Twenty-four female Beagle dogs sourced from Marshall Bioresources (NY, USA) were assigned to the study. At the time of randomization, they were between 1.5 and 2.9 years of age with body weights ranging from 9.3 to 11.6 kg. Each animal was identified with a unique ear tattoo and was implanted with a microchip. Bitches were in at least their second heat period when they were mated. Animals were mated 2 or 3 times, with one day interval between matings. Breeding occurred over 12 days; a different male was used for each female; for a total of 24 different males. Successful mating was defined as an observation of a tie. Males used for mating were proven breeders at least 12 months of age and confirmed healthy. Six females per group were mated to have minimum five pregnancies per group. Animals were tested for pregnancy 29 to 35 days after mating. When all six dams within a group were confirmed pregnant, one dam was randomly selected to be released from the study, so that five pregnant females per group continued on the study. Animals were assigned to cages/pens in order of ascending animal mating number for blinding purposes. Dams were housed individually in European cages (approximately 5 by 6 feet, i.e. 1.5 m by 1.8 m) equipped with an automatic watering system. About two weeks prior to parturition, a nesting box was offered to each dam. Parturition occurred over 11 days. After parturition, on post-partum day 1 (PPD 1), the size of litters with more than six puppies was adjusted by removing extra puppies by random selection to yield six puppies; the removed puppies were cross fostered on segregate dams. Similarly, on PPD 4, the size of each litter was further adjusted to 4 puppies/litter, with at least one male and one female per litter. Starting on PPD 49, a daily period of separation of the puppies from their mother was introduced. The duration was gradually increased until complete separation on PPD 56. While separated from their mother during the weaning process, the puppies had access to a bowl of electrolyte solution. At weaning on PPD 56, one puppy per sex per litter was randomly retained for the 26 weeks growth phase of the study. Dams and puppies not selected to continue on the growth phase of the study were transferred to the Citoxlab animal colony. After weaning, puppies selected for the study were pair housed in stainless steel dog cages (approximately 3 by 3 feet, i.e. 0.9 m by 0.9 m) equipped with an automatic watering system. They were separated for 6–8 hours daily to acquire individual food consumption data starting two weeks after weaning. All cages were clearly labeled with cage cards, indicating a study number, animal tattoo number, blinded animal assignment number, sex and replicate. All animals were transferred to the Citoxlab animal colony upon completion of the study.

The animal room environment was set to maintain a relative humidity of 50 ± 20%; a light dark cycle of 12 hours /12 hours, and 10–15 air changes per hour. The room temperature was maintained at 21 ± 3°C from the study start until approximately 2 weeks prior to parturition when it was increased to maintain 24 ± 3°C until PPD 42. From PPD 43 (of the youngest litter) until the end of the study, the temperature was returned to 21 ± 3°C. Municipal tap water, exposed to ultraviolet light and purified by reverse osmosis at Citoxlab North America only, was provided to the animals *ad libitum*, via automatic water system. A bowl of water was also placed in the cage until weaning.

### Animal assignment and blinding procedures

Each dam was assigned an animal number determined randomly. Females were assigned to different dietary groups based on an assignment number. Blinding in the study was applied to the identity of the test and the control diets assigned to each experimental group (i.e., Groups 1 through 4). Only animal numbers (e.g., 1, 2, etc.) were maintained in the electronic data capturing system. Therefore, there were no indicators of animal assignment to treatment groups in the data capturing system until the study was unblinded after cessation of treatment. Personnel involved in the data collection were blinded to the experimental treatments. The access to the document with the animal assignment table was restricted to unblinded personnel which included a study director, a clinical pathologist, the pharmacy and veterinary staff, and the departmental team leaders. The unblinded personnel did not participate in direct data collection. This exception to the blinding requirement was necessary to permit adequate monitoring and quality checks during the study and to ensure animal welfare.

### AOCED and diets

Dosing of AOCED was confirmed with batch records and analytically, using combined DHA+EPA as a reference for AOCED addition level. The nominal content of DHA+EPA in AOCED is 50% but can vary depending on the production lot. The AOCED lot VY00015572 used for the test diets preparation contained DHA at 42.7 area% of total fatty acids (400.3 mg/g oil) and EPA at 13.6 area% of total fatty acids (124.7 mg/g oil). Therefore, the total DHA+EPA content was 56.3 area% or 525 mg/g oil. Arachidonic acid was present at 1.99 area% of total fatty acids. The peroxide value of the oil was 0.30 meq/kg and p-anisidine value was 2.9.

Experimental diets were manufactured at Kansas State University (Manhattan, KS, USA) in May 2017. The diets, including the control, were formulated by DSM Nutritional Products to meet the AAFCO Dog Food Nutrient Profile for Growth and Reproduction (AAFCO, 2016). The dry kibble diets were manufactured using a Wenger X20 extruder and then dried in a Wenger dryer. They were cooled before coating with flavors and a mixture of chicken fat and fish oil (control), or a mixture of chicken fat and AOCED (AOCED-containing diets). The diets were made isocaloric with the control diet. The nutrient levels of the experimental diets were confirmed to meet or exceed the AAFCO minimums but not exceed the maximums via analysis by Eurofins (Des Moines, IA). Ingredient composition of the Base Kibble is presented in Table 1 while the test diets’ composition is shown in Table 2 and S1 Table. AOCED levels were verified by measurements of the DHA and EPA content in the diets. Samples from each formulation were collected upon manufacture and shipped to Eurofins for analysis. Diets were coded with letters, numbers and colored labels to protect blinding.

**Table 1:** Composition of Base Kibble (on as is basis).

Ingredient Name	%
Chicken by-product meal	26.0
Corn	25.4
Dried eggs	14.9
Brewers rice	12.2
Milo	5.3
Wheat	5.3
Dog Palatability Enhancer (AFB International, MO, USA)	3.2
Corn gluten meal	2.0
Beet pulp	1.9
Dicalcium Phosphate	1.6
Potassium chloride	0.8
Salt	0.7
Vitamin premix^1^	0.3
Calcium carbonate	0.2
Verdilox GT Dry^2^	0.1
Mineral premix^3^	0.1
Choline chloride	0.1
Total	100

^1^Contains vitamin E supplement, niacin supplement, thiamine mononitrate, D-calcium pantothenate, vitamin A supplement, pyridoxine hydrochloride, riboflavin supplement, vitamin D3 supplement, vitamin B12 supplement, folic acid, biotin, mineral oil, pea fiber, and calcium carbonate.

^2^Verdilox GT Dry is commercial antioxidant and flavoring product from Kemin Industries, Inc (Des Moines, Iowa).

^3^Contains ferrous sulfate, zinc oxide, manganous oxide, copper sulfate, sodium selenite, cobalt carbonate, calcium carbonate, mineral oil, and ethylenediamine dihydriodide.

**Table 2:** Fat composition in the test diets (on as is basis).

Diet	Control	Low dose AOCED	Mid dose AOCED	High dose AOCED
Ingredients	% of Diet			
Base kibble	94	94	94	94
Chicken fat	5.70	5.25	4.52	3.08
Fish oil (14% EPA, 12% DHA)	0.27	0	0	0
AOCED	0	0.72	1.45	2.89
Verdilox GT Liquid	0.03	0.03	0.03	0.03
Total	100	100	100	100

Diets were stored at room temperature throughout the study. Samples from each diet were collected in duplicates at the study start (Day 1 of dosing), at two-month intervals throughout the study, and at the end of the study. One set of samples was shipped after each collection point at ambient temperature to DSM analytical lab (Dartmouth, Canada) for measurements of DHA and EPA concentrations and peroxide value determination. The retention samples were stored at room temperature at Citoxlab until the study completion for a re-analysis. Fatty acid analysis of dietary samples collected throughout the study confirmed acceptable levels of EPA+DHA which ensured proper AOCED dosing of animals. Recovery values were generally above 80% of the EPA+DHA values determined by Eurofins upon diets manufacture and specified in the certificates of analysis. Peroxide values were below 10 mEq/Kg throughout the duration of the study.

### Administration of diets

All females were fed a commercial standard diet (ProPet Puppy Performance 30/22; St. Mary, Ohio) containing minimum 30% of crude protein and minimum 22% of crude fat for at least one week prior to receiving the control diet from Study Days -7 through -1 (acclimation period/ pre-treatment period). Feeding of experimental diets started on Day 1 defined as the day of the first successful mating. From Study Day 1 onward, the control group continued to receive the control diet, while three other groups received food with 0.75%, 1.5% or 3.0% of AOCED (Table 3).

**Table 3:** Study design.

Study Group	Dosage Level (X of targeted dose)	Target Dosage Level (% of AOCED in the diet on DM basis)	Actual DHA+EPA content* (% in the diet As Is)	DHA+EPA content (% in the diet on DM basis) **	Number of Animals		
					F_0_ Generation (dams)^a^	F_1_ Generation (puppies)^b^	
					F	M(Spare)	F(Spare)
Control	0	0	0.11%	0.12%	5	4(1)	4(1)
AOCED Low Dose	½X	0.75%	0.41%	0.44%	5	4(1)	4(1)
AOCED Mid Dose	1X	1.5%	0.77%	0.83%	5	4(1)	4(1)
AOCED High Dose	2X	3.0%	1.55%	1.69%	5	4(1)	4(1)

* Values presented as a sum of DHA and EPA (Weight %) per Certificate of Analysis.

** Calculated values based on dry matter basis (adjusted for moisture content).

^a^ Six females per group were mated to have at least 5 females per group confirmed pregnant at GD 29–35.

^b^ Two puppies per litter (a male and a female) were randomly selected, the spare was randomly selected from any litter within each group.

M: male; F: female

Dams received approximately 400 g of food per day during the acclimation, then about 600 g per day from mating to parturition, and 900 g per day from parturition until weaning.

Starting at 5 weeks of age and until weaning, puppies were provided with a feeding pan (approximately 300 g of feed). During that period, the daily food intake was calculated for the dam plus its litter (i.e.: dam’s bowl, plus feeding pan) since the dam also had access to the feeding pan. After weaning, puppies selected for the study continued on their mothers’ respective diets (approximately 400 g per day/dog for a 6–8 hours daily feeding period) for the additional 26 weeks until they reached 34 weeks of age. Puppies remained pair-housed for feeding during the first two weeks post-weaning to confirm ability to thrive, and then were individually housed for feeding for the remainder of the study.

### Clinical observations

Clinical signs were recorded twice daily throughout the study. The observations included evaluation of general appearance, fur condition, activity, and other indicators of changes in animal health and behavior, including gait and mobility. Litter checks were performed once daily (concurrently with a clinical sign activity) until PPD 56. A detailed physical examination was performed on each animal on the day of estrous and on the day of treatment initiation, and monthly thereafter. The last physical examination was performed on the day prior to or on the day of the animal release from the study. During the examination, animals were observed while in a cage for behavior, general appearance, coordination, and then removed from the cage for close evaluation of general condition of the whole body including head, thoracic, and abdominal areas through examination and palpation. Body temperature, heart rate and respiratory rate (counts per minute) were also evaluated.

For all puppies, height and head circumference were measured weekly starting on PPD 7.

In addition, a detailed examination (including body temperature, heart rate and respiratory rate) was performed on all puppies selected to continue on the growth phase starting on PPD 63 (one-week post-weaning) and monthly thereafter, including within three days prior to their release from the study. This evaluation included assessment of the general body condition, autonomic and central nervous system, somatomotor activity, gait, skin/fur condition, lymph nodes, eyes, mucus membranes, respiration, behavior, circulatory system, state of hydration, and lungs, bladder, reproductive system and gastrointestinal auscultation and/or palpation. Additional observations were performed as deemed necessary.

Body weights and body weight changes were recorded for all dams on the day of estrous and on the day of treatment initiation, and weekly thereafter. Puppies were weighed every 3 or 4 days, i.e., PPD 0, 4, 7, 10, 14, 17, 21, etc., until weaning, and weekly thereafter. Body weight measurements were also performed on dams and puppies on the day prior to their release from the study.

### Food consumption

Individual food intake was measured daily throughout the study, starting at initiation of feeding the reference/the control diet (3–14 days prior to treatment start). In addition to the daily food consumption, achieved intakes were calculated on a weekly basis. Any spilled food was also weighed daily for each cage, unless contaminated with liquid or solid material (e.g. water, urine, feces, etc.). The amount of contaminated food (excluded from the spillage measured value) was visually estimated and documented.

The average of daily individual achieved intake of the test item in mg/kg body weight per week was calculated for both generations based on daily food consumption using the formula:

$$\frac{Dietary\;concentration\;(\frac{mg}{kg})\times food\;consumption\;(kg)}{Start\;week\;body\;weight\;(kg)}$$

For dams with puppies from 5 weeks of age until weaning (with feeding pan, including puppies body weight), and for the first two weeks post-weaning (2 puppies/cage), the following formula was used:

$$\frac{Dietary\;concentration\;(\frac{mg}{kg})\times food\;consumption\;(\frac{kg}{cage})}{Start\;week\;body\;weight\;(\frac{kg}{cage})}$$

### Parturition and litter observations

Dams were monitored for signs of parturition at least 3 times daily starting from gestation day (GD) 55. Body temperature was measured twice daily (12 ± 1 hour apart) starting on GD 55 until the first signs of parturition were observed. Body temperature was also measured daily from GD 52 to 54 for baseline acquisition. The time of onset and completion of delivery was recorded for each animal when possible. The day when parturition was completed was termed as PPD 0. Any signs of difficulty in parturition were recorded.

At birth, number of live, dead and malformed puppies was recorded. Gross abnormalities (if any) were recorded. In addition, number of male and female puppies in each litter was determined by visually judging the anogenital distance and/or presence of a vulva or penis. Recordings of live and dead puppies continued daily from PPD 0 until weaning on PPD 56.

### Clinical pathology

Blood samples were collected via venipuncture of the jugular vein for hematology and clinical chemistry from all dams once during pretreatment and at weaning. In addition, coagulation and urinalysis were performed on all puppies during Weeks 13 and 34; while hematology and clinical chemistry evaluation was performed at weaning and every two months thereafter for a total of 4 occasions during the growth phase. Blood samples for hematology (1.3 mL) were collected into tubes containing K_3_-EDTA as anticoagulant. Samples for coagulation (1.0 mL) were collected into tubes containing citrate as anticoagulant while clotting activator gel was in the tubes used for collection of clinical chemistry samples (1.1 mL). Animals were fasted overnight (approximately 8–9 hours) before blood samples collection.

### Dose formulation analysis and peroxide value determination

Fatty acid concentrations in the dietary samples collected during the study were analyzed at DSM analytical lab (Dartmouth, Canada). Samples were lyophilized and then homogenized with a food grinder. The samples were dissolved in equal parts toluene and 1.5N HCl in methanol. Tricosanoin was added as an internal standard (NuCheck Prep, Elysian, MN). Samples were heated for 2 hours at 80°C and neutralized with 6% sodium carbonate solution. Aliquots of the organic layer containing the FAMEs were analyzed by using a gas chromatograph equipped with a FAMEWAX 30m x 0.32 mm x 0.25 μm column (Restek, Bellefonte, PA). Fatty acids were identified by comparison with a standard mixture of fatty acids (GLC 714, NuCheck Prep, Elysian, MN). Fatty acid composition was expressed as a weight percent (% w/w) of total identified fatty acids in a sample.

Recoveries of a total EPA + DHA content in diet samples collected throughout the study were calculated by dividing the measured EPA + DHA concentration at each time point by the EPA + DHA concentration reported by Eurofins (Des Moines, IA) in the initial Certificates of Analysis for each respective diet.

The FoodLABfat analyzer was used to analyze peroxide values in dog diet samples by a colorimetric assay to measure peroxide oxidized Fe^2+^ ions as a marker of lipid oxidation. Back-up samples were analyzed by Eurofins (Des Moines, IA) at the study completion.

### Fatty acid analysis of plasma

Blood samples (3 mL each) were collected from dams during the pretreatment period for a baseline, after 4 to 5 weeks of dietary treatment and at weaning. Blood samples (2 mL each) were also collected from all selected weaned puppies at weaning and during Weeks 13 and 34, at approximately the same time on each occasion. Samples were collected by jugular venipuncture into tubes containing K_2_-EDTA as anticoagulant and separated to plasma and red blood cells. Plasma was shipped on dry ice to OmegaQuant (Sioux Falls, SD, USA) for fatty acid analysis. The fatty acid analysis was done by gas chromatography with flame ionization detection (GC/FID). Plasma samples were directly trans esterified with 14% boron trifluoride, toluene, methanol; 35:30:35 v/v/v. Tricosanoin was added as an internal standard, (NuCheck Prep, Elysian, MN). Samples were heated 45 minutes at 100°C. The Fatty Acid Methyl Esters (FAMEs) were extracted with hexane (EMD Chemicals, USA) and washed with water. An aliquot of the hexane layer was analyzed using a GC-2010 Gas Chromatograph (Shimadzu Corporation, Columbia, MD) equipped with a SP-2560, 100-m fused silica capillary column (0.25 mm internal diameter, 0.2 um film thickness; Supelco, Bellefonte, PA). Fatty acids were identified by comparison with a standard mixture of fatty acids (GLC-782, NuCheck Prep, Elysian, MN) and expressed as concentrations, μg of fatty acid per mL of plasma.

### Statistical analysis

The statistical analyses for this study were designed with reference to the Guidance for Industry #226: “Target Animal Safety Data Presentation and Statistical Analysis” of the Center of Veterinary Medicine (CVM), a division of the US Food and Drug Administration (FDA). As recommended by the guidance, no statistical adjustments for multiplicity were applied to avoid false negative findings and the significance level for comparisons against the control group was α = 0.1 for safety endpoints. In the context of toxicology, this is a statistically conservative approach because it increases the probability of identifying adverse effects, and thus reduces the risk of overlooking any potential safety issues. The interpretation of the results was based both on statistical significance and clinical relevance, as recommended by the guidance.

The statistical analyses were independently performed for the following three periods: gestation, pre and post weaning. The treatment group was used as a fixed effect in all models.

Analysis of variance (ANOVA), analysis of covariance (ANCOVA) as well as repeated measures ANOVA/ANCOVA and linear mixed models were employed, depending on the available number of timepoints within a period and whether a baseline measurement was available.

For parameters with only one timepoint within a period, with only one sex and without baseline value, a one-way ANOVA was applied, e.g. for pre-treatment body-weights of dams. If a baseline, i.e. pre-treatment value was also available then ANCOVA was applied, including the baseline as covariate, e.g. for clinical pathology measurements of dams at weaning. Analysis on pre-weaning puppies included only those selected to continue on study after litters were equalized.

For parameters with more than one timepoint within a period, repeated measures equivalents of the above methods were employed to take into account potential effects over time. The fixed effects in the models were the treatment group, the time and their interaction, with the animal as subject and, when applicable, the pre-treatment value as covariate. For the efficacy parameters (EPA and DHA plasma levels), mixed effects models with random intercept by animal were used.

Three-way repeated measures ANOVA were used for longitudinal analyses on puppies, where males and females had to be differentiated, and when more than one follow-up timepoint within a period was available, e.g. for body weight of puppies post-weaning. The fixed effects in the model were the treatment group, sex, time and their two-way and three-way interactions with the animal as subject. For the pre-weaning period, the statistical unit was the puppy nested within litter.

Further details and a list of the type of models that were employed for each parameter are provided in S1 Appendix.

Statistical analyses were performed in SAS version 9.2 and 9.3, R version 3.4.3 and Minitab [16, 17].

## Results

Clinical signs observed in maternal females during the gestation and the lactation periods of the study were mainly related to variations in stool consistency, reported as soft/loose/liquid stool and reduced/absent fecal output. The signs were observed in all groups, including the control, and were not considered related to the consumption of the experimental diets. Fecal changes are common in laboratory-housed dogs, especially during pregnancy and parturition. There were no diet related effects on body temperatures, heart rates and respiratory rates during the gestation and the lactation periods. Individual variations in these parameters were considered incidental since similarly observed in the control animals, were transient and/or reflected the normal inter-animal variation in this species.

There was no AOCED related effect on food consumption in females during the gestation and the lactation phases of the study. Average daily food consumption over gestation was 179 ± 32, 198 ± 17, 179 ± 22, and 177 ± 25 g/day in the control, AOCED low dose, AOCED mid dose, and AOCED high dose groups, respectively. During the eight weeks of lactation (Post Parturition Day, PPD 0–55), these values were 512 ± 189, 538 ± 170, 540 ± 197, 549 ± 184 g/day.

**Fig 1:**
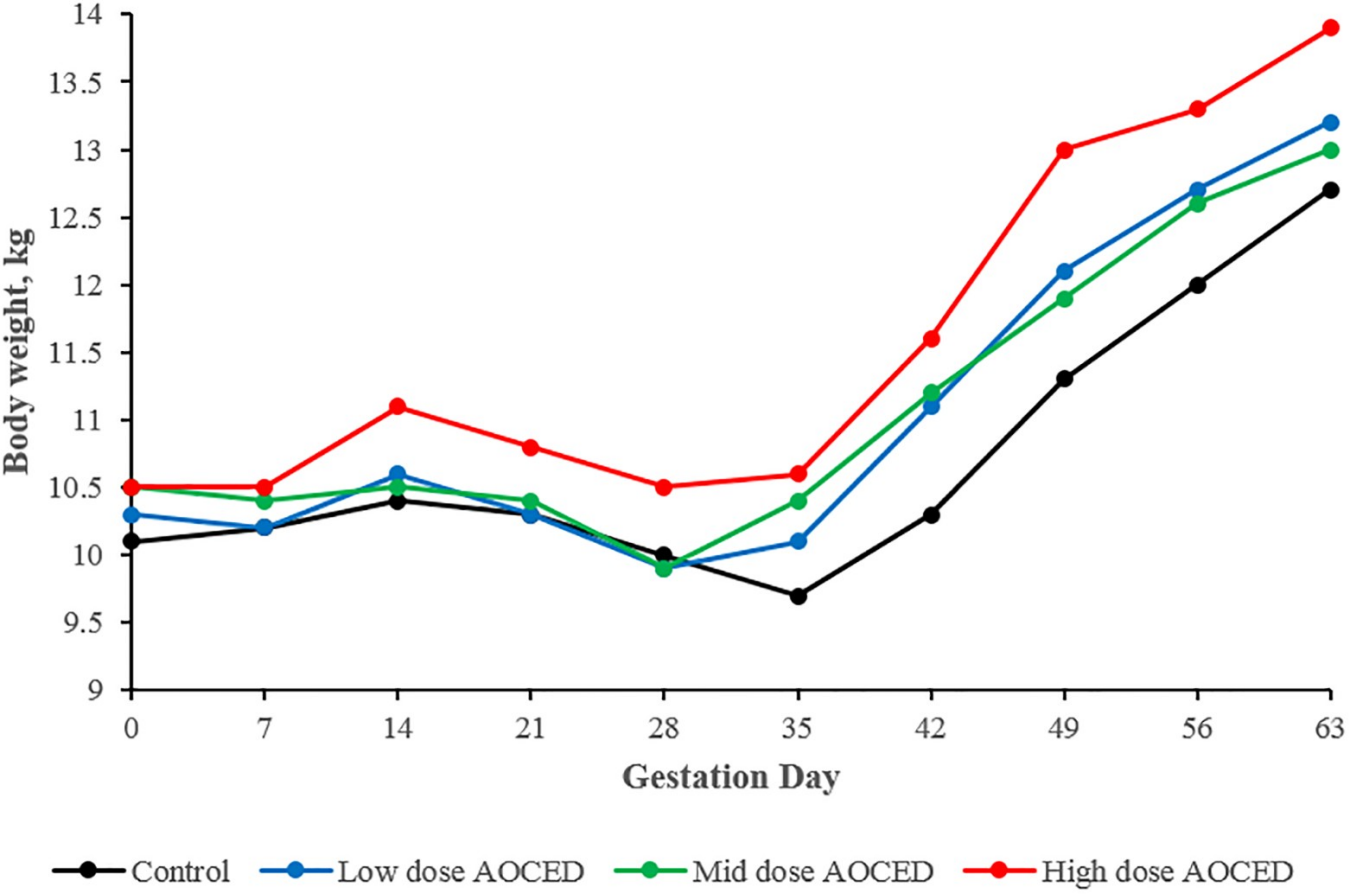
Body weights of females during gestation. Values are shown as group means (n = 5).

There were no statistically-significant differences between groups in body weights during gestation but there was a trend to higher body weights and body weight gains in all AOCED groups compared to the control (Fig 1). During the lactation period (PPD 0–56), average body weights of females were comparable across groups: 10.0 ± 0.6, 10.0 ± 0.8, 10.2 ± 0.7, and 10.4 ± 1.2 kg in the control, AOCED low-dose, AOCED mid-dose, and AOCED high- dose groups, respectively. Similarly, average body weight changes from PPD0 to PPD 56 were not affected by the test diets: 0.11 ± 0.03, 0.02 ± 0.06, 0.04 ± 0.07, and 0.13 ± 0.06 kg in the same group order as above.

No changes in hematology and clinical chemistry parameters were observed in dams in the AOCED groups when compared to those in the control at the completion of the lactation phase (S2 and S3 Tables). Reduction of the mean cholesterol level was noted in the AOCED high dose group but was not considered adverse.

There were no dams found dead or that had to be euthanized during the study. However, three dams were released from the study and transferred to the Citoxlab animal colony shortly after completion of the parturition process since they were left with no live puppies. All other dams displayed normal maternal behavior.

One dam in the AOCED high-dose group expelled a still-born fetus on GD 55, and the remaining fetuses on GD 57. The dam displayed decreased activity, markedly reduced appetite, an elevated body temperature (38.9°C), greenish-black liquid feces, tremors, decreased capillary refill time and retching. Intravenous administration of fluids and antibiotics was started immediately due to a suspected infection. One dam in the AOCED low-dose group experienced no problems during the parturition process and had nine live puppies; however, by PPD 2, they were cannibalized or not nursed by the dam. Similarly, there were no issues with the parturition of one dam in the mid-dose group; but all puppies from that litter died by PPD 5 due to a lack of nursing, which was confirmed by the absence of milk in the stomach observed at necropsy. These three cases were not considered related to the administration of the test diets given their dissimilar etiology and were likely incidental in nature.

There was no AOCED related effects on litter size or viability index at birth (Table 4). The percentage of live puppies at birth varied from 89% in the control group to 100% in the high dose group. Mean body weights of puppies at birth were overall similar across groups with a trend to higher values in the low- and mid-dose AOCED groups both in males and females (Table 4). There were no detrimental AOCED effects on the puppy viability before PPD 4 and survival indices up to PPD 56. On the contrary, the viability and survival indices were generally higher in AOCED groups, especially in the high dose group where only a single death was observed out of 38 born puppies (Table 4). After PPD 4, there was only one mortality observed across all groups: the last live puppy of the mid-dose dam mentioned above died on PPD 5, leading to a release of the dam from the study.

**Table 4:** Dams’ reproductive parameters and puppies’ viability.

Group	Number of Litters*	Litter size on PPD0*	PPD0 Viability Index (%)	PPD1-4 Survival Index (%)	Mean BW Males on PPD0 (g)	Mean BW Females on PPD0 (g)
Control Diet	5	7.6	89.3	83.5	227 ± 35	208 ± 35
Low dose AOCED	4 (5)	8.3 (8.4)	96.1	96.3	247 ± 36	225 ± 43
Mid dose AOCED	4 (5)	7.5 (7.2)	96.4	84.6	270 ± 20	250 ± 35
High dose AOCED	4	9.5	100	98.9	235 ± 15	214 ± 11

Litter size is an average number of puppies born per litter on PPD0. PPD0 viability index is a percentage of puppies born live on PPD 0. PPD 1–4 Survival Index is a percentage of puppies survived by PPD 4. PPD 0 BW—average body weight of born puppies on PPD 0. PPD—post parturition day. Mean body weight (BW) data are shown as mean ± SD.

*Values in brackets () include litters from one dam in Low dose group and one dam in Mid dose group which were ultimately released from the study due to mortality of their entire litter later on.

There were no clinical observations in puppies from birth (PPD 0) to weaning (PPD 56) that were considered attributable to the dietary treatments. Clinical signs included skin changes (bruises, dryness, redness, lump, scabbed and wounded), tail kinked, firm/soft swelling of flank or cervical dorsal areas, and fur thin/dull/stained. Most adverse clinical observations of decreased activity, weakness, recumbency, body thinness, cold to touch, signs of dehydration, etc., were noted prior to PPD 5, generally in puppies that did not survive. These observations were considered incidental since they occurred across all groups including dogs in the control groups, are common in puppies of this age and species, and showed no relationship to dose levels in incidence or severity.

At weaning on PPD 56, one puppy from AOCED high dose group exhibited red buccal discharge, retching, labored and increased respiration, as well as being cold to touch. These clinical signs were observed shortly after blood sampling and therefore were considered procedure-related. Due to the animal condition, it was released from the study on PPD 56 and replaced by a spare from the same litter to continue to the growth phase of the study.

**Fig 2:**
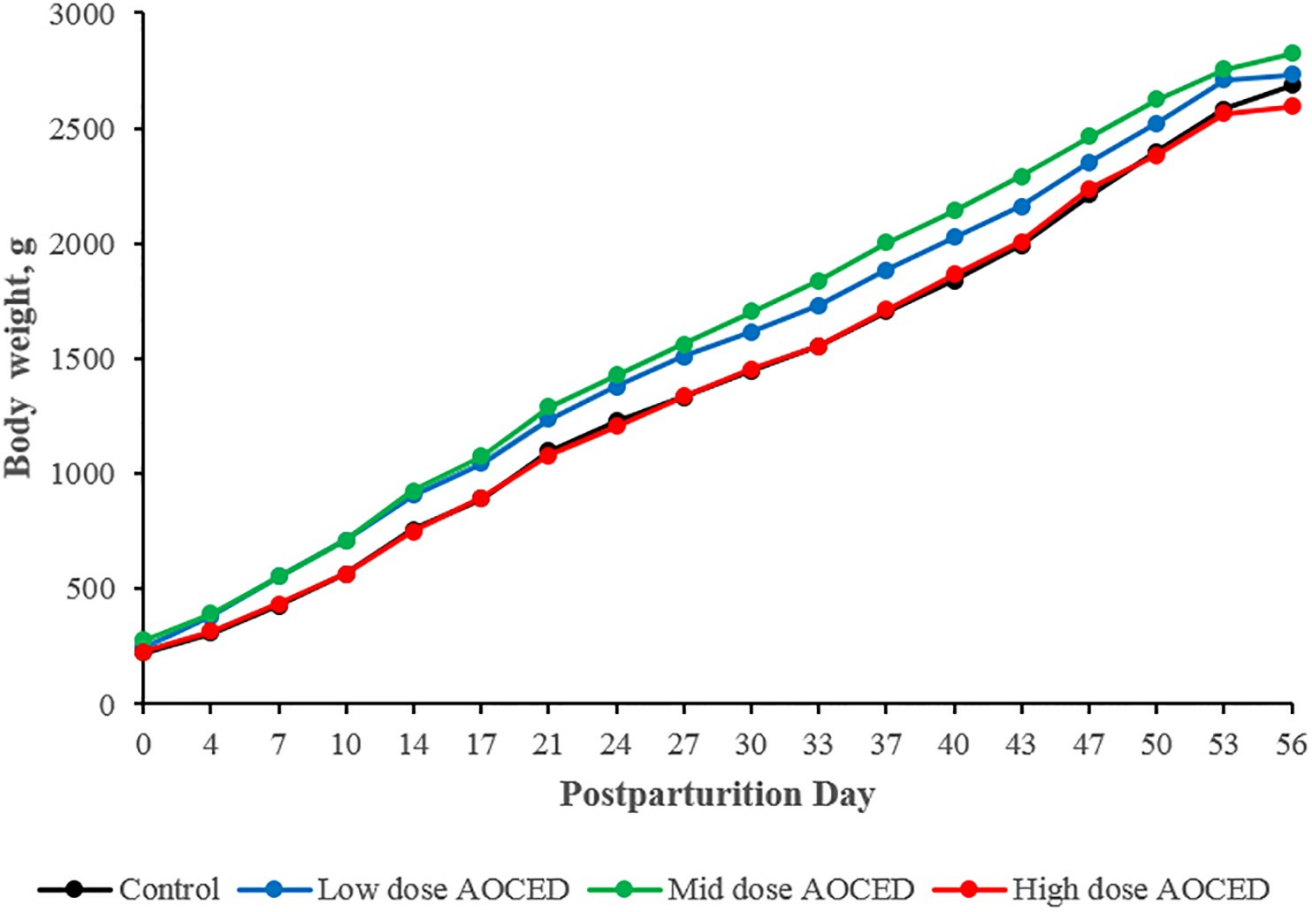
Body weights of puppies before weaning. There were no statistically significant differences between males and females, therefore, genders were combined. Values are shown as group means (n = 10).

Mean body weights of F1 puppies from birth to weaning were increased in the low- and mid-dose groups compared to the control in both sexes, reaching statistical significance on most occasions (Fig 2). Given the lack of a dose-response, these increases were considered incidental and not related to the administration of AOCED.

There were no AOCED related effects on the height or height changes of F1 puppies from the first measurement on PPD 7 until weaning that were considered attributable to AOCED diets. Variations in height or height changes including some higher values in the low- and mid-dose groups that resulted in statistically significant differences from those in the control group on some occasions were considered incidental since there was no dose related pattern.

During the lactation period from the first measurement on PPD 7 onward, the mean litter head circumference of puppies in the low- and mid-dose groups was increased compared to that in the control in both sexes, reaching statistical significance on most measurement occasions, especially during the first half of the lactation phase (S1 Fig). The differences mainly disappeared by weaning, and the mean values were similar among all groups. Given the lack of a dose-response and transient nature, these increases were considered incidental and not related to the AOCED diets.

There were no F1 mortalities in any group during the growth phase of the study which lasted for 26 weeks after weaning. Further, there were no AOCED-related clinical observations during this phase. Some sporadic changes in stool consistency occurred across all groups including the control and were, therefore, considered incidental. No differences among groups were observed in body temperature, heart rate and respiratory rate during the growth period.

**Fig 3:**
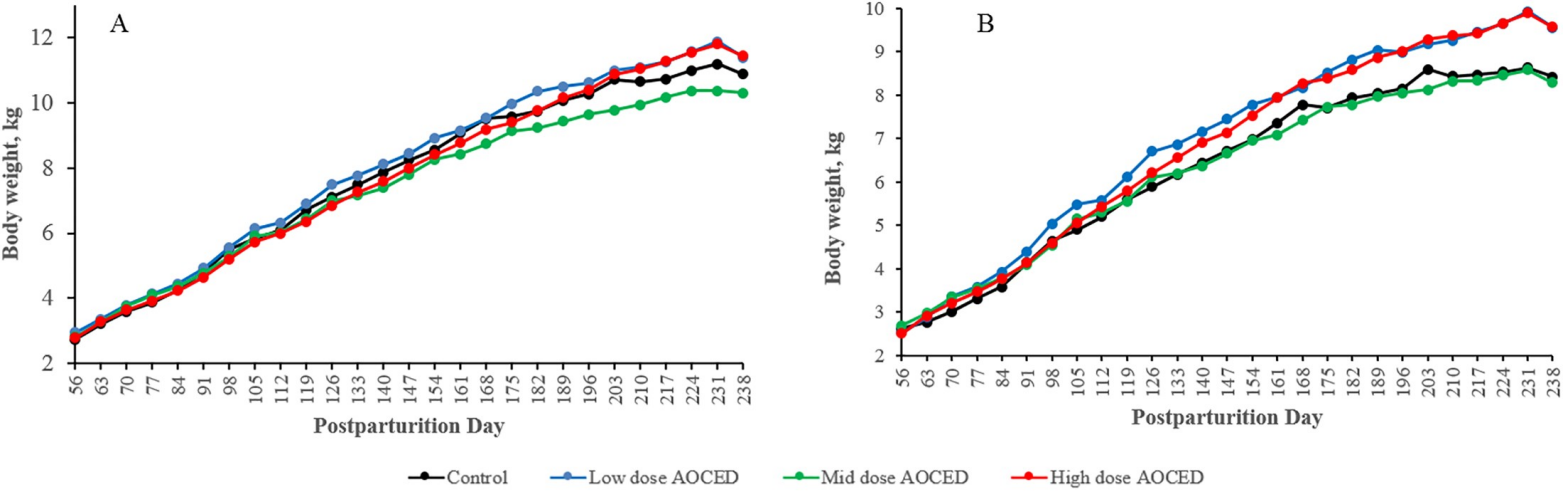
Body weights of puppies (A- males; B—females) after weaning. Values are shown as group means (n = 5).

Body weights of puppies after weaning are shown in Fig 3. From weaning (PPD 56) onward, mean body weights of the low- and high-dose puppies were increased compared to the control in both sexes, reaching statistical significance on most occasions from PPD 182 onward. Consequently, the overall mean body weight gains for the whole growth phase (PPD 238 compared to PPD 56) in the low- and high dose groups were generally above the control mean. However, the same was not true for the mid-dose group. In males of the mid-dose group, mean body weights in the second half of the growth phase were even lower than those in the control (Fig 3B). Given the lack of a dose-response, the body weight changes were considered incidental and not related to the administration of AOCED.

**Fig 4:**
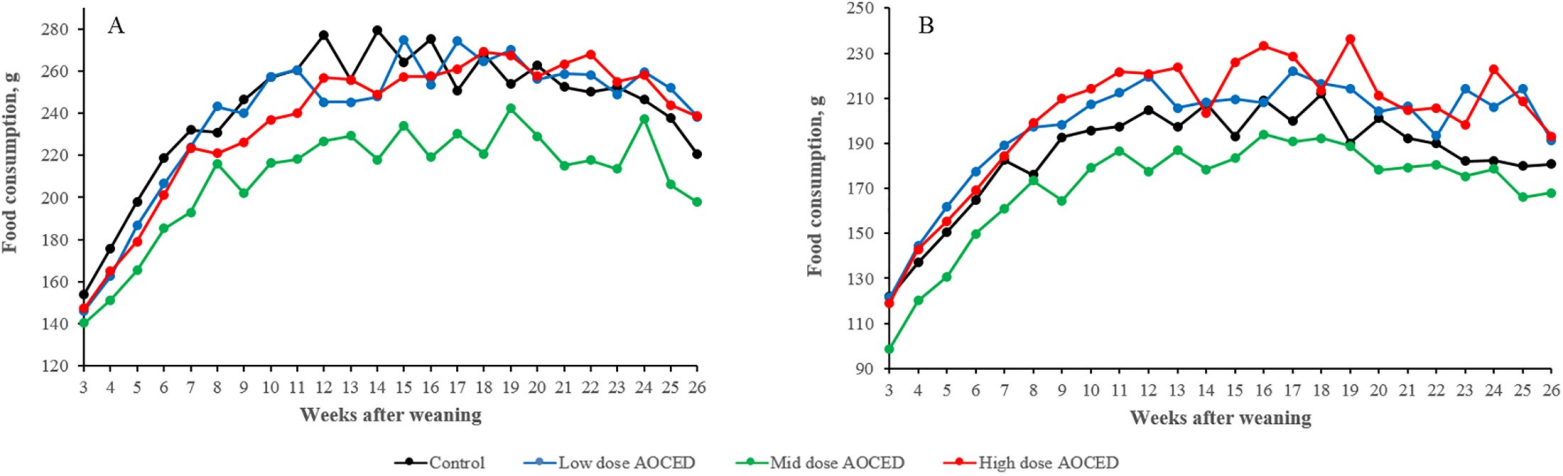
Food consumption of puppies (A—males; B—females) after weaning. Values are shown as group means (n = 5) in grams per dog per day. Statistically significant differences were observed between the mid-dose and control males on Weeks 7, 12, 14 and 18 (p<0.1). Statistically significant differences were observed between the mid-dose and control females on Weeks 3 and 14 (p<0.1), and between the high-dose and control groups on Weeks 15, 19, and 24 (p<0.1).

The group-mean food consumption after weaning was comparable across all groups, including the control, in both sexes. There was a trend toward reduced food consumption in the mid-dose animals in both sexes, but differences did not reach statistical significance on most occasions (Fig 4). In females of the low- and high dose groups, mean food consumption values were generally higher than that of the control, but again without statistical significance on most occasions (Fig 4B).

No changes in hematology, clinical chemistry and coagulation parameters were noted in puppies fed the test diets compared to those fed the control diet at the study completion (Tables 5–7). Differences were observed in some hematology parameters over the different time points during the study, but they were transient and consistent with normal biological variability in growing dogs and thus considered incidental. No changes were noted in coagulation parameters during the study at both measurements (Table 7). Creatinine values increased over time in all animals with primary increases by PPD 168 and peaking at PPD 224. This was expected due to increased muscle mass during the growth period and was noted in all groups, including the control.

**Table 5:** Hematology values at study completion (PPD 224) following AOCED exposure starting *in utero* until puppies reach 6 months after weaning.

Parameter	Sex	Control	Low Dose AOCED	Mid Dose AOCED	High Dose AOCED
Red blood cells count (x10^-12^/L)	M	7.06±0.53	7.34±0.56	6.84±0.21	7.06±0.54
	F	7.64±0.48	7.53±0.32	7.07±0.31	7.13±0.41
Hemoglobin (g/L)	M	162.5±11.8	163.3±12.0	156.0±7.0	163.0±12.0
	F	179.0±17.5	171.3±7.1	163.3±11.8	162.0±8.8
Hematocrit (L/L)	M	0.49±0.03	0.50±0.03	0.47±0.03	0.49±0.04
	F	0.54±0.04	0.52±0.02	0.49±0.03	0.49±0.02
Mean corpuscular volume (fL)	M	69.6±1.1	67.4±1.9	68.7±2.1	69.3±0.5
	F	70.3±0.7	68.9±1.2	69.2±1.2	68.9±2.5
Mean corpuscular hemoglobin, MCH (pg)	M	23.0±0.7	22.2±0.6	22.9±0.6	23.1±0.3
	F	23.5±0.6	22.8±0.5	23.1±0.8	22.8±0.9
MCH concentration (g/L)	M	330.8+4.8	329.8+4.6	332.3+5.9	333.5+2.4
	F	333.8+5.7	330.0+2.6	333.0+6.3	330.5+4.7
Red cell distribution width (%)	M	13.7±0.7	13.6±0.5	12.9±0.8	13.0±0.9
	F	13.6±0.5	13.6±0.7	12.9±0.7	13.2±0.7
Platelet count (x10^-9^/L)	M	250.8+52.2	224.5+33.7	234.8+45.7	255.8+10.5
	F	187.0±20.8	219.8±44.6	214.5±19.4	236.8±23.8
White blood cells count (x10^-9^/L)	M	8.85±1.52	9.91±1.3	10.12±2.9	9.32±0.89
	F	9.11±1.7	9.57±0.3	7.44±1.0	8.14±1.1
Neutrophils count (x10^-9^/L)	M	5.04±1.30	5.77±0.80	5.94±2.07	5.26±0.83
	F	4.79±0.95	5.21±0.53	3.99±0.66	4.19±1.02
Lymphocyte count (x10^-9^/L)	M	2.98±0.28	3.28±0.47	3.23±0.77	3.20±0.11
	F	3.30±0.79	3.53±0.29	2.78±0.48	3.29±0.15
Monocytes count (x10^-9^/L)	M	0.38±0.08	0.42±0.01	0.49±0.10	0.50±0.06
	F	0.59±0.17	0.43±0.10	0.33±0.04	0.42±0.03
Eosinophils count (x10^-9^/L)	M	0.35±0.17	0.33±0.05	0.35±0.09	0.28±0.09
	F	0.30±0.06	0.28±0.09	0.24±0.17	0.16±0.03
Basophiles count (x10^-9^/L)	M	0.07±0.02	0.06±0.01	0.07±0.05	0.05±0.03
	F	0.09±0.02	0.09±0.03	0.06±0.04	0.06±0.03
Large unstained cells (x10^-9^/L)	M	0.035±0.02	0.043±0.02	0.035±0.01	0.028±0.01
	F	0.048±0.02	0.038±0.01	0.030±0.01	0.028±0.01
Reticulocytes count (x10^-9^/L)	M	47.2±24.8	51.5±7.3	41.0±10.9	42.7±17.7
	F	74.9±28.9	63.9±26.4	44.6±18.3	44.6±17.5

Values are given as mean ± SD (n = 5 in each group). No statistically significant differences were observed among groups (p<0.1).

**Table 6:** Clinical chemistry values at the study completion (PPD 224) following AOCED exposure starting *in utero* until puppies reach 6 months after weaning.

Parameter	Sex	Control	Low Dose AOCED	Mid Dose AOCED	High Dose AOCED
AST (U/L)	M	28.0 ± 0.8	29.8 ± 2.4	29.5 ± 2.6	36.3 ± 1.7
	F	28.8 ± 2.2	31.0 ± 2.8	26.5 ± 1.7	34.8 ± 2.6
ALT (U/L)	M	37.3 ± 3.9	30.3 ± 5.6	37.3 ± 14.9	62.3 ± 21.8
	F	39.5 ± 6.6	42.0 ± 3.6	37.0 ± 13.0	63.0 ± 29.6
ALP (U/L)	M	113.8 ± 61.1	98.3 ± 17.1	113.3 ± 56.6	137.5 ± 19.2
	F	98.5 ± 21.2	79.8 ± 27.1	114.5 ± 50.3	112.3 ± 4.3
BUN (mg/dL)	M	5.10 ± 0.7	5.43 ± 0.6	5.40 ± 0.4	6.28 ± 0.8
	F	4.88 ± 0.8	5.58 ± 0.8	5.28 ± 0.6	5.65 ± 0.7
CREA (μmol/L)	M	51.3 ± 4.6	52.8 ± 4.5	58.3 ± 5.4	52.0 ± 7.9
	F	55.8 ± 4.6	56.0 ± 6.0	58.8 ± 5.1	46.8 ± 4.6
CHOL (mmol/L)	M	6.22 ± 0.36	6.24 ± 0.75	5.56 ± 0.26	5.68 ± 0.64
	F	6.13 ± 0.54	5.67 ± 1.38	5.80 ± 0.84	5.30 ± 0.63
TRIG (mmol/L)	M	0.37 ± 0.08	0.46 ± 0.08	0.43 ± 0.10	0.39 ± 0.06
	F	0.43 ± 0.09	0.45 ± 0.07	0.42 ± 0.10	0.40 ± 0.05
GLUC (mmol/L)	M	5.68 ± 0.38	5.45 ± 0.35	5.63 ± 0.51	5.70 ± 0.24
	F	5.80 ± 0.6	5.23 ± 0.26	5.48 ± 0.28	5.55 ± 0.39
TP (g/L)	M	54.3 ± 1.8	53.2 ± 0.8	54.3 ± 2.2	57.3 ± 2.3
	F	55.9 ± 0.9	53.7 ± 1.5	54.7 ± 2.9	54.9 ± 1.4
ALB (g/L)	M	34.4 ± 2.6	34.9 ± 1.7	36.5 ± 1.7	37.7 ± 1.1
	F	36.3 ± 1.3	36.4 ± 2.2	37.5 ± 2.5	36.8 ± 0.8
GLOB (g/L)	M	19.9 ± 1.4	18.4 ± 1.5	17.7 ± 2.0	19.6 ± 1.4
	F	19.7 ± 1.7	17.3 ± 1.2	17.2 ± 0.9	18.1± 1.0
A/G	M	1.74 ± 0.2	1.92 ± 0.3	2.09 ± 0.3	1.92 ± 0.1
	F	1.86 ± 0.2	2.12 ± 0.3	2.18 ± 0.2	2.04 ± 0.1
CK (U/L)	M	195 ± 22	199 ± 26	211 ± 116	262 ± 194
	F	162 ± 21	201 ± 40	143 ± 12	196 ± 40
AMYL (U/L)	M	700 ± 98	847 ± 143	786 ± 89	683 ± 111
	F	706 ± 119	709 ± 93	591 ± 95	634 ± 141
TBA (μmol/L)	M	1.75 ± 0.8	1.65 ± 0.9	4.77 ± 2.4	2.10 ± 0.6
	F	2.53 ± 2.1	4.53 ± 3.1	3.57 ± 2.5	3.93 ± 1.3

Values are given as mean ± SD (n = 5 in each group). No statistically significant differences were observed among groups (p<0.1).

AST, Aspartate Aminotransferase; AST, Alanine Aminotransferase; ALP, Alkaline Phosphatase; BUN, Blood Urea Nitrogen; CREA, Creatinine; CHOL, Cholesterol; TRIG, Triglycerides; GLUC, Glucose; TP, Total Protein; ALB, Albumin; GLOB, Globulin; CK, Creatine Kinase; AMYL, amylase; TBA, Total Bile Acids.

**Table 7:** Coagulation data of puppies at Weeks 13 and 34 of age.

Parameter	Sex	Week 13			Week 34		
		PT, sec	APTT, sec	Fib, g/L	PT, sec	APTT, sec	Fib, g/L
Control	M	7.78±0.26	27.30±1.99	2.38±0.28	7.90±0.29	22.28±2.20	2.02±0.43
	F	7.88±0.17	26.60±2.31	2.03±0.10	7.88±0.24	21.63±2.57	2.00±0.58
AOCED Low Dose	M	7.73±0.19	25.35±2.26	2.22±0.10	8.03±0.35	22.88±4.20	1.83±0.24
	F	7.78±0.36	24.35±2.40	2.20±0.33	8.28±0.35	23.58±5.14	1.59±0.19
AOCED Mid Dose	M	7.73±0.26	24.35±1.47	2.15±0.23	8.20±0.32	21.48±2.66	2.02±0.42
	F	7.55±0.13	23.98±1.64	1.95±0.17	8.53±0.17	22.23±1.55	1.35±0.17
AOCED High dose	M	7.78±0.28	25.88±2.02	2.13±0.22	8.25±0.34	21.23±2.71	1.63±0.08
	F	7.93±0.38	23.63±3.06	1.89±0.13	8.05±0.49	22.15±2.06	1.78±0.37

Values are given as mean ± SD (n = 4 per group). APTT—Activated Partial Thromboplastin Time; Fib—Fibrinogen; PT—Prothrombin Time. No statistically significant differences were observed among groups (p<0.1).

**Fig 5:**
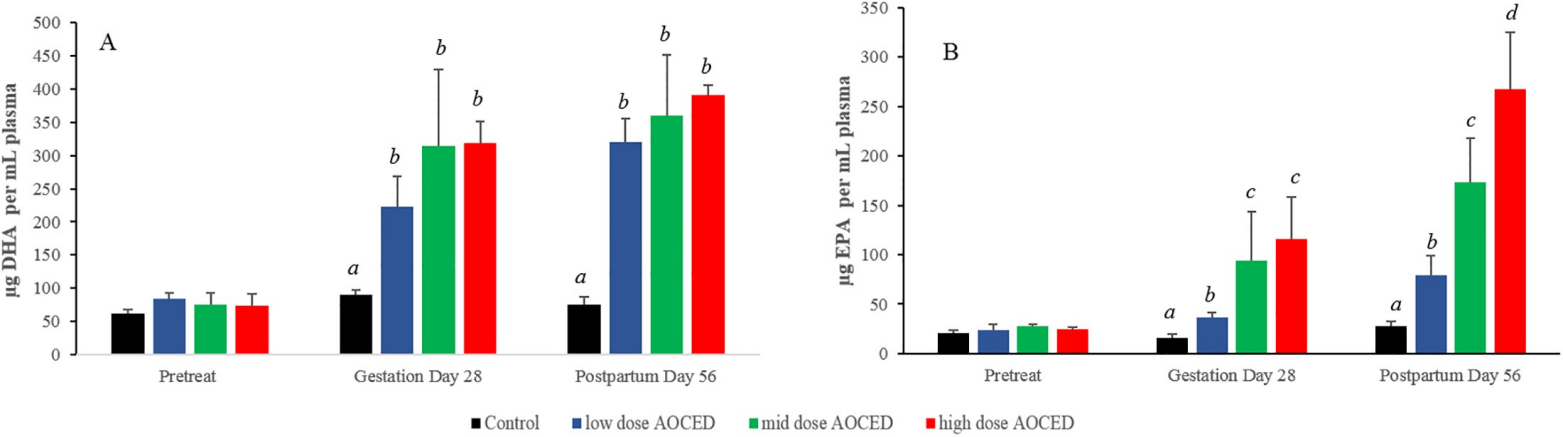
Plasma DHA (A) and EPA (B) levels in dams. Values are given as Mean + SD and expressed as μg/mL, n = 4 in each group. ^*a*,*b*,*c*,*d*^ signify between-group differences at a timepoint (p<0.05).

The administration of AOCED in the diets resulted in plasma DHA and EPA increases in maternal dogs after four weeks of feeding (GD 28) and continued feeding until the end of lactation period (Fig 5). No baseline differences in plasma DHA and EPA concentrations were observed among the groups (Pretreat in Fig 5). Significant plasma DHA increases were observed in all test diet groups at both GD 28 and PPD 56 compared to that in the control group, but differences between dose groups did not reach statistical significance (Fig 5A). Plasma EPA increases were more dose-dependent, as their levels in the mid- and high-dose groups were higher than those in the low-dose at GD 28 and with a clear dose-response at PPD 56 (Fig 5B).

A linear mixed model which included a random intercept by subject revealed overall significant effects of time (p<0.001), dose (p<0.001) and dose-time interaction (p<0.001) on both DHA and EPA plasma increases in dams. All treatment groups increased significantly more than the control group with AOCED administration (p<0.01) but plasma EPA increases between GD 28 and PPD 56 were more pronounced (Fig 5B).

In contrast to increases in DHA and EPA, plasma arachidonic acid levels declined by the end of lactation in AOCED mid- and high-dose groups compared to the control although differences reached statistical significance in the high-dose group only (p<0.05). The concentrations were 1000 ± 136, 964 ± 131, 809 ± 185 and 641 ± 94 μg/mL in the control, AOCED low-dose, AOCED mid-dose, and AOCED high-dose groups, respectively.

**Fig 6:**
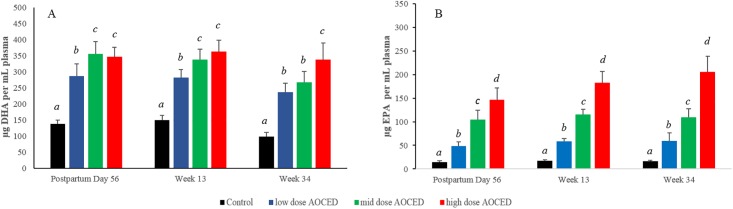
Plasma DHA (A) and EPA (B) levels in puppies (combined genders). Values are given as Mean + SD and expressed as μg/mL, n = 8 in each group. ^*a*,*b*,*c*,*d*^ signify between-group differences at a timepoint (p<0.05).

In puppies, the administration of AOCED in the diets started in utero, and thus resulted in plasma DHA and EPA increases compared to those in the control at all timepoints (Fig 6). There were no differences between genders in plasma DHA and EPA levels at any measured timepoint, therefore, sexes were combined. At weaning on PPD 56, the puppies’ plasma DHA and EPA levels in the test groups were significantly higher than those in the control group, indicating that the maternal fatty acid status affected their offspring. For plasma DHA, there were statistically significant differences between the low- and mid-dose AOCED groups but not between the mid- and high-dose groups at weaning and Week 13 of age (Fig 6A). At the study completion when puppies were 34 weeks of age, plasma DHA levels did not differ significantly between the low- and mid-dose AOCED groups but were different compared to the high-dose level (Fig 6A). The increases in plasma EPA levels were dose dependent across all three dose levels at all measured timepoints (Fig 6B).

A linear mixed model which included a random intercept by subject revealed overall significant effects of time (p<0.001), dose (p<0.001) and dose-time interaction (p<0.001) for both DHA and EPA plasma increases in puppies. Plasma DHA and EPA levels generally remained flat over the growth period between weaning and the completion of the study, although plasma EPA concentrations in the high-dose AOCED group continued to raise modestly with time (Fig 6).

Plasma arachidonic acid (ARA) levels in the high-dose AOCED group were lower compared to that in the control at weaning (p<0.05). Plasma ARA levels were 853 ± 59, 775 ± 79, 754 ± 118 and 479 ± 72 μg/mL in the control, AOCED low-dose, AOCED mid-dose, and AOCED high-dose groups, respectively. By Week 13, the differences with the control reached statistical significance in all AOCED groups (data not shown). At the study completion at Week 34, the concentrations were 1091 ± 114, 954 ± 130, 810 ± 76 and 745 ± 109 μg/mL in the control, AOCED low- dose, AOCED mid-dose, and AOCED high-dose groups, respectively, with the mid- and high- dose levels being significantly lower than the control (p<0.05).

The average of daily individual achieved intake of AOCED in mg/kg body weight per week, calculated based on daily food consumption and weekly body weight measurements, was overall dose proportional during all phases of the study: the gestation and the lactation in dams and the growth phase in puppies (Table 8). The achieved AOCED intake in the mid-dose group was ~250, 640 and 440 mg/kg body weight/day during the gestation, the lactation, and the growth phase, respectively (Table 8).

**Table 8:** Achieved AOCED intake.

Dietary group	Dams Gestation (GD 0–62)	Dams Lactation (PPD 0–55)	Puppies Combined Post-weaning Weeks 1–2	Puppies Males Post-weaning Weeks 3–26	Puppies Females Post-weaning Weeks 3–26
Low dose AOCED	138.3	319.4	314.0	231.7	236.6
Mid dose AOCED	248.6	638.6	568.5	435.5	440.2
High dose AOCED	474.9	1243.9	1310.0	959.5	975.8

Values represent average daily AOCED intake over a period (mg/kg body weight/day).

## Discussion

A comprehensive study in Beagle dogs was undertaken to provide evidence of safety and bioavailability of Algal Oil Containing EPA and DHA (AOCED) for reproduction, development and growth, at the dog early life stages as well at later in life. The administration of AOCED at dietary levels of 0.75%, 1.5% and 3.0%, which corresponded to doses of DHA and EPA of 0.44%, 0.83% and 1.69%, to female dogs, starting at mating and continued throughout gestation and lactation, was well tolerated by the dogs and did not affect their overall health and physiological parameters. Food consumption, body weights and body weight gains of AOCED-treated animals were comparable to those of the control group (Fig 1). There were no changes in hematology and clinical chemistry parameters in females fed AOCED diets compared to the control other than reduced cholesterol levels in the high-dose group (S2 and S3 Tables). This was expected due to known lipid-lowering effects of omega-3 fatty acids [18] and was observed in dogs administered omega-3 algal oils before [19].

There were no detrimental AOCED effects on litter size or puppy viability and survival indices (Table 4). Moreover, the viability and survival indices were generally higher in the AOCED dietary groups compared to those in the control group, confirming that DHA and EPA are needed for optimal fetal and neonatal development [4]. Further, puppy body weights, height and head circumference before weaning were generally increased in the low- and mid-dose AOCED groups compared to those in the control (Table 4, Fig 2 and S1 Fig). Interestingly, there were no differences in growth parameters observed in the high-dose AOCED group compared to the control; the increases were limited to the low- and mid-dose groups. The apparent limitations in beneficial effects of AOCED on early dog development by doses up to 1.5% in the diet could be related to modulating effects of DHA and EPA on arachidonic acid levels. Arachidonic acid (ARA) is needed for adequate growth and development during perinatal nutrition along with DHA [20]. In the human studies with pre-term infants, growth parameters were affected negatively in a group consuming formula supplemented with marine oil [21]. This effect was attributed to the decline in ARA blood levels observed in the marine oil group compared to the standard formula control [22]. To achieve a balance between omega-3 and omega-6 fatty acids, commercial infant formulae are typically supplemented with both DHA and ARA.

A competitive relationship between the long chain omega-3 and omega-6 fatty acids has been reported in animal studies as well [3, 23, 24]. Dietary DHA and EPA compete with ARA for incorporation into cell membrane phospholipids [25]. Accordingly, supplemented DHA and EPA allow for the partial replacement of ARA in the lipids of blood cells and organ cells thereby reducing the ARA content and the amount of ARA available to form metabolites [26, 27]. We observed declines in ARA tissue levels with administration of high doses of algal oils across multiple species including rats, dogs and humans [15, 28, 29]. The AOCED lot used in the present study contained 2.0% ARA and 56.3% of combined DHA+EPA as area% of total fatty acids (see Methods for details). Plasma ARA reductions were observed in the high-dose AOCED group both in the dams at the end of lactation period and in the puppies at weaning (see Results for details). Noteworthy, no detrimental effects on growth parameters were observed even in the high-dose group compared to the control, indicating that there was sufficient amount of ARA in the diet to sustain an adequate growth. However, the beneficial effects of AOCED diets on growth parameters observed in the low- and mid-dose AOCED groups were absent in the high-dose group, i.e. the growth was similar to the control rather than superior (Fig 2 and S1 Fig).

After weaning, body weights of puppies during the 26 weeks growth phase were increased compared to the control in the low- and high-dose animals of both genders, with more pronounced differences observed in females (Fig 3). In the mid-dose group males, body weights in the second half of the growth phase were lower compared to the control (Fig 3A). The body weight changes generally correlated with the observed alterations in food consumption in these animals (Figs 3 and 4) and was not attributed to AOCED administration.

No changes in hematology and clinical chemistry parameters were noted in the test diet groups during the growth phase (Tables 5 and 6). Importantly, no AOCED effects were noted on standard coagulation parameters (Table 7). It was suggested that long-term consumption of EPA- and DHA-enriched diets may decrease platelet activation and function although no effects on platelet aggregation were observed in dogs [12]. In the present study, platelet numbers did not differ between dietary groups in both generations and were within reference values for these animals (Table 5 and S2 Table). Further, coagulation parameters were not affected by the AOCED dietary treatment (Table 7). In addition, no increases in petechiae or hematomas developing from the handling and blood collection during the study were observed in the AOCED groups. Therefore, it was considered that animals across all groups had normal platelet activity.

DHA and EPA from AOCED were clearly bioavailable as demonstrated by the dose-dependent increases in levels of these fatty acids in plasma of dogs in both generations (Figs 5 and 6). The essentially linear plasma DHA profiles observed in both dams during gestation and lactation and puppies during growth indicate that DHA levels had approached the steady state shortly after GD 28 and increased only incrementally thereafter (Figs 5A and 6A). This supports published human and dog studies which reported that plasma DHA concentrations typically plateau in about a month after the start of DHA supplementation [28, 29]. Noteworthy, plasma DHA increases were more dose-dependent in puppies than in dams (Figs 5A and 6A). In contrast, plasma EPA levels increased generally in a dose-dependent manner in both generations (Figs 5B and 6B) and continued to raise with time in dams (Fig 5B). However, plasma EPA increases over time were more modest in puppies (Fig 6B).

In the present study, average daily AOCED intakes in the mid-dose group were approximately 250 mg/kg body weight/day during gestation, ~640 mg/kg body weight/day during lactation, and about 440 mg/kg body weight/day during growth, based on the food consumption and body weight data. These levels are higher than those used in most reported dog studies [11, 12, 30]. A recommended allowance for the combination of DHA and EPA for dog growth and reproduction was set by the National Research Council publication on Nutrient Requirements of Dogs and Cats at 0.5 g/kg diet on dry matter basis, equivalent to 0.05 wt% [4]. The safe upper limit for the combined DHA and EPA was established at 11 g/kg diet or 1.1 wt% [4]. The diets in our study contained nominal total DHA and EPA amounts of 0.12% (the control), 0.44% (the low-dose AOCED), 0.83% (the mid- dose AOCED) and 1.69% (the high-dose AOCED); therefore, all exceeded the recommended allowance and even the safe upper limit in the high-dose diet. However, there were no treatment-related adverse effects noted at any dose level in any phases of the study.

The results of the present study demonstrate the safety and bioavailability of Algal Oil Containing EPA and DHA at dietary levels up to 3.0%, equivalent of 30 g/kg diet, on a dry matter basis.

## Supporting information

S1 Table Chemical composition of the experimental diets. (DOCX)

S2 Table Dams’ hematology values following AOCED exposure starting at mating until the end of lactation. Values are given as mean ± SD (n = 5 in control; n = 4 in AOCED groups).
(DOCX)

S3 Table Dams’ clinical chemistry values following AOCED exposure starting at mating until the end of lactation. Values are given as mean ± SD (n = 5 in control; n = 4 in AOCED groups).
(DOCX)

S4 Table Dams’ clinical chemistry values raw data. (XLSX)

S5 Table Puppies’ clinical chemistry values raw data. (XLSX)

S6 Table Puppies head circumference pre-weaning raw data. (XLSX)

S7 Table Dams’ body weights during gestation raw data. (XLSX)

S8 Table Puppies’ body weights during lactation raw data. (XLSX)

S1 Fig Head circumference of puppies before weaning. There were no statistically significant differences between males and females, therefore, genders were combined. Values are shown as group means (n = 10).
(TIFF)

S1 Appendix Statistical analysis. (DOCX)
