# Correction: Safety of a novel feed ingredient, Algal Oil containing EPA and DHA, in a gestation-lactation-growth feeding study in Beagle dogs

**DOI:** 10.1371/journal.pone.0246487

**Published:** 2021-01-28

**Authors:** Irina Dahms, Eileen Bailey-Hall, Erin Sylvester, Audrey Parenteau, Shiguang Yu, Alexios Karagiannis, Franz Roos, Jon Wilson

In the Animals and housing subsection of the Materials and methods, there is an error in the sixteenth sentence of the first paragraph. The correct sentence is: After parturition, on post-partum day 1 (PPD 1), the size of litters with more than six puppies was adjusted by removing extra puppies by random selection to yield six puppies; the removed puppies were euthanized by intracardiac injection of sodium pentobarbital solution at 240 mg/mL and dose volume based on the standard operating procedures of the testing facility.
