# Supplementary material for: Safety of a novel feed ingredient, Algal Oil containing EPA and DHA, in a gestation-lactation-growth feeding study in Beagle dogs
Source: PLoS One. 2019 Jun 3;14(6):e0217794. doi: 10.1371/journal.pone.0217794 (PMC6546231; doi:10.1371/journal.pone.0217794)
Supplement: S1 Table — (DOCX) [file pone.0217794.s001.docx]

**S1 Table. Chemical composition of the experimental diets.**

|  | Control | Low dose AOCED | Mid dose AOCED | High dose AOCED |
| --- | --- | --- | --- | --- |
| Moisture* | 7.4% | 6.9% | 7.1% | 8.3% |
| Crude Protein | 30.89% | 32.00% | 32.29% | 32.85% |
| Arginine | 1.84% | 1.93% | 1.98% | 2.02% |
| Histidine | 0.67% | 0.69% | 0.70% | 0.70% |
| Isoleucine | 1.30% | 1.33% | 1.35% | 1.32% |
| Leucine | 2.45% | 2.52% | 2.55% | 2.57% |
| Lysine | 1.88% | 1.90% | 1.86% | 1.89% |
| Methionine | 0.73% | 0.75% | 0.76% | 0.73% |
| Cystine | 0.45% | 0.45% | 0.47% | 0.45% |
| Phenylalanine | 1.36% | 1.41% | 1.43% | 1.42% |
| Tyrosine | 0.86% | 0.91% | 0.99% | 1.01% |
| Threonine | 1.22% | 1.26% | 1.27% | 1.30% |
| Tryptophan | 0.36% | 0.35% | 0.38% | 0.38% |
| Valine | 1.62% | 1.68% | 1.69% | 1.67% |
| Crude fat | 17.53% | 17.59% | 17.66% | 18.75% |
| Linoleic acid | 3.60% | 3.52% | 3.36% | 3.02% |
| Alpha-linolenic acid | 0.17% | 0.16% | 0.16% | 0.14% |
| Arachidonic acid | 0.17% | 0.19% | 0.22% | 0.26% |
| EPA+DHA | 0.12% | 0.44% | 0.83% | 1.69% |
| Calcium | 1.72% | 1.64% | 1.52% | 1.36% |
| Phosphorus | 1.24% | 1.21% | 1.15% | 1.09% |
| Potassium | 0.98% | 0.96% | 0.90% | 0.82% |
| Sodium | 0.600% | 0.590% | 0.557% | 0.513% |
| Chloride | 1.32% | 1.27% | 1.14% | 1.04% |
| Magnesium | 0.109% | 0.108% | 0.107% | 0.105% |
| Iron | 540 ppm | 519 ppm | 465 ppm | 408 ppm |
| Copper | 26 ppm | 26 ppm | 23 ppm | 22 ppm |
| Manganese | 87 ppm | 90 ppm | 79 ppm | 77 ppm |
| Zinc | 282 ppm | 275 ppm | 262 ppm | 243 ppm |
| Iodine | 3.50 ppm | 3.19 ppm | 3.19 ppm | 2.90 ppm |
| Selenium | 0.84 ppm | 0.93 ppm | 0.82 ppm | 0.77 ppm |
| Vitamin A (IU/kg) | 34,665 | 31,364 | 22,978 | 23,555 |
| Vitamin D (IU/kg) | 2,959 | 2,256 | 2,659 | 2,246 |
| Vitamin E (IU/kg) | 367 | 359 | 319 | 272 |
| Thiamine | 42.3 ppm | 41.7 ppm | 40.8 ppm | 41.5 ppm |
| Riboflavin | 27.6 ppm | 19.1 ppm | 19.8 ppm | 8.6 ppm |
| Pantothenic acid | 43.4 ppm | 42.7 ppm | 40.3 ppm | 41.9 ppm |
| Niacin | 232 ppm | 230 ppm | 249 ppm | 201 ppm |
| Pyridoxine | 20.2 ppm | 20.1 ppm | 18.7 ppm | 18.8 ppm |
| Folic acid | 4.20 ppm | 4.10 ppm | 3.78 ppm | 4.35 ppm |
| Vitamin B12 | 0.120 ppm | 0.116 ppm | 0.125 ppm | 0.123 ppm |
| Choline | 3,595 ppm | 3,420 ppm | 3,600 ppm | 3,315 ppm |

* Moisture content was used to calculate values on a dry matter basis.
