# Supplementary material for: Safety of a novel feed ingredient, Algal Oil containing EPA and DHA, in a gestation-lactation-growth feeding study in Beagle dogs
Source: PLoS One. 2019 Jun 3;14(6):e0217794. doi: 10.1371/journal.pone.0217794 (PMC6546231; doi:10.1371/journal.pone.0217794)
Supplement: S2 Table — Values are given as mean ± SD (n = 5 in control; n = 4 in AOCED groups). (DOCX) [file pone.0217794.s002.docx]

**S2 Table. Dams’ hematology values following AOCED exposure starting at mating until the end of lactation**.

| Parameter | Control | Low Dose AOCED | Mid Dose AOCED | High Dose AOCED |
| --- | --- | --- | --- | --- |
| RBC (x10^-12^/L) | 7.20 + 0.61 | 7.50 + 0.86 | 7.01 + 0.49 | 7.09 + 0.30 |
| HGB (g/L) | 171.2 + 11.4 | 174.0 + 20.2 | 158.5 + 12.1 | 162.8 + 12.6 |
| HCT (L/L) | 0.51 + 0.04 | 0.52 + 0.06 | 0.47 + 0.04 | 0.48 + 0.03 |
| MCV (fL) | 70.4 + 1.3 | 69.7 + 0.6 | 67.7 + 2.5 | 68.4 + 3.8 |
| MCH (pg) | 23.8 + 0.5 | 23.2 + 0.4 | 22.6 + 0.9 | 22.9 + 1.4 |
| MCHC (g/L) | 338.4 + 2.6 | 333.5 + 3.7 | 334.3 + 2.2 | 335.8 + 2.4 |
| RDW (%) | 12.4 + 0.9 | 12.4 + 0.5 | 13.2 + 1.4 | 13.2 + 1.6 |
| PLT (x10^-9^/L) | 324 + 60 | 302 + 74 | 306 + 37 | 332 + 56 |
| WBC (x10^-9^/L) | 7.52 + 1.7 | 7.69 + 0.4 | 9.93 + 1.3 | 8.08 + 1.4 |
| NEUT (x10^-9^/L) | 4.01 + 0.9 | 5.00 + 0.3* | 6.42 + 0.7** | 4.99+ 1.3* |
| LYMPH (x10^-9^/L) | 2.29 + 0.7 | 1.87 + 0.7 | 2.06 + 0.5 | 2.23 + 0.5 |
| MONO (x10^-9^/L) | 0.40 + 0.08 | 0.31 + 0.05 | 0.53 + 0.14 | 0.44 + 0.13 |
| EOS (x10^-9^/L) | 0.75 + 0.5 | 0.46 + 0.3 | 0.83 + 0.6 | 0.38+ 0.1 |
| BASO (x10^-9^/L) | 0.034 + 0.01 | 0.038 + 0.01 | 0.035+ 0.01 | 0.025 + 0.01 |
| LUC (x10^-9^/L) | 0.032 + 0.01 | 0.020 + 0.00 | 0.035 + 0.01 | 0.020 + 0.01 |
| NEUT (%) | 53.8 + 6.5 | 65.2 + 7.7 | 64.9 + 3.7 | 61.2 + 8.1 |
| LYMPH (%) | 30.1 + 3.8 | 24.0 + 7.1 | 20.8 + 3.9 | 28.2 + 7.3 |
| MONO (%) | 5.5 + 1.5 | 3.9 + 0.5 | 5.4 + 1.5 | 5.4 + 1.2 |
| EOS (%) | 9.7 + 6.5 | 5.9 + 3.1 | 8.2 + 4.8 | 4.7 + 1.1 |
| BASO (%) | 0.42 + 0.16 | 0.48 + 0.13 | 0.35 + 0.06 | 0.33 + 0.10 |
| LUC (%) | 0.44 + 0.22 | 028 + 0.05 | 0.35 + 0.06 | 0.28 + 0.05 |
| RETIC (x10^-9^/L) | 29.4 + 5.9 | 30.4 + 18.5 | 32.2 + 3.7 | 26.5 + 18.3 |
| RETIC (%) | 0.42 + 0.11 | 0.38 + 0.21 | 0.48 + 0.05 | 0.38 + 0.22 |

Values are given as mean + SD (n=5 in control; n=4 in AOCED groups).

* indicates statistically significant difference from Control (p < 0.1)

** indicates statistically significant difference from Control (p < 0.05)

RBC, red blood cells count; HGB, hemoglobin; HCT, hematocrit; MCV, mean corpuscular volume; MCH, mean corpuscular hemoglobin; MCHC, mean corpuscular hemoglobin concentration; RDW, red cell distribution width; PLT, platelet count; WBC, white blood cells count; NEUT, neutrophils; LYMPH, lymphocytes; MONO, monocytes; EOS, eosinophils; BASO, basophils; LUC, large unstained cells; RETIC, reticulocytes.
