# Supplementary material for: Safety of a novel feed ingredient, Algal Oil containing EPA and DHA, in a gestation-lactation-growth feeding study in Beagle dogs
Source: PLoS One. 2019 Jun 3;14(6):e0217794. doi: 10.1371/journal.pone.0217794 (PMC6546231; doi:10.1371/journal.pone.0217794)
Supplement: S3 Table — Values are given as mean ± SD (n = 5 in control; n = 4 in AOCED groups). (DOCX) [file pone.0217794.s003.docx]

**S3 Table**. **Dams’ clinical chemistry values following AOCED exposure starting at mating until the end of lactation.**

| Parameter | Control | Low Dose AOCED | Mid Dose AOCED | High Dose AOCED |
| --- | --- | --- | --- | --- |
| AST (U/L) | 26.6 + 3.2 | 27.3 + 4.6 | 24.3 + 1.7 | 32.3 + 6.1 |
| ALT (U/L) | 32.4 + 10.6 | 42.8 + 22.9 | 31.0 + 5.5 | 35.8 + 3.3 |
| ALP (U/L) | 97.6 + 33.4 | 99.3 + 22.9 | 91.5 + 30.6 | 80.8 + 9.9 |
| BUN (mmol/L) | 5.54 + 0.92 | 5.45 + 1.39 | 6.25 + 2.01 | 5.68 + 1.13 |
| CREA (μmol/L) | 46.0 + 4.2 | 47.8 + 10.6 | 48.8 + 16.1 | 39.3 + 3.1 |
| CHOL (mmol/L) | 6.94 + 0.85 | 6.82 + 0.64* | 6.99 + 2.2 | 5.67 + 0.87** |
| TRIG (mmol/L) | 0.51 + 0.06 | 0.41 + 0.07 | 0.39 + 0.04 | 0.43 + 0.11 |
| GLUC (mmol/L) | 5.44 + 0.18 | 4.73 + 0.15* | 5.75 + 0.40** | 5.43 + 0.61 |
| TP (g/L) | 61.3 + 2.9 | 60.0 + 1.5 | 61.6 + 1.6 | 61.0 + 2.6 |
| ALB (g/L) | 34.4 + 1.8 | 35.6 + 1.3 | 35.6 + 1.7 | 34.6 + 1.6 |
| GLOB (g/L) | 26.9 + 1.6 | 24.5 + 1.6 | 25.9 + 0.5 | 26.4 + 1.6 |
| A/G | 1.28 + 0.08 | 1.46 + 0.13 | 1.37 + 0.08 | 1.31 + 0.09 |
| Ca (mmol/L) | 2.55 + 0.04 | 2.55 + 0.17 | 2.54 + 0.08 | 2.50 + 0.01 |
| Phos (mmol/L) | 1.10 + 0.14 | 1.24 + 0.32 | 1.10 + 0.11 | 1.09 + 0.12 |
| Na (mmol/L) | 146.2 + 1.1 | 147.3 + 1.5 | 147.5 + 1.9 | 146.3 + 0.5 |
| K (mmol/L) | 4.60 + 0.18 | 4.71 + 0.47 | 4.64 + 0.43 | 4.70 + 0.12 |
| Cl (mmol/L) | 106.6 + 2.0 | 108.1 + 0.8 | 107.1 + 2.6 | 107.0 + 1.1 |
| CK (U/L) | 153.8 + 32.6 | 155.8 + 51.4 | 112.3 + 15.8 | 170.3 + 42.9 |
| AMYL (U/L) | 391.8 + 27.5 | 389.5 + 43.8 | 475.3 + 97.7 | 780.8 + 426.7 |
| TBA (μmol/L) | 1.70 + 0.28 | 1.15 + 0.21 | 4.00 + 4.24 | 1.80 + 0.18 |

Values are given as mean + SD (n=5 in control; n=4 in AOCED groups).

* indicates statistically significant difference from Control (p < 0.1)

** indicates statistically significant difference from Control (p < 0.05)

AST, Aspartate Aminotransferase; AST, Alanine Aminotransferase; ALP, Alkaline Phosphatase; BUN, Blood Urea Nitrogen; CREA, Creatinine; CHOL, Cholesterol; TRIG, Triglycerides; GLUC, Glucose; TP, Total Protein; ALB, Albumin; GLOB, Globulin; CK, Creatine Kinase; AMYL, amylase; TBA, Total Bile Acids.
