# Supplementary figures and images for: Safety of a novel feed ingredient, Algal Oil containing EPA and DHA, in a gestation-lactation-growth feeding study in Beagle dogs
Source: PLoS One. 2019 Jun 3;14(6):e0217794. doi: 10.1371/journal.pone.0217794 (PMC6546231; doi:10.1371/journal.pone.0217794)

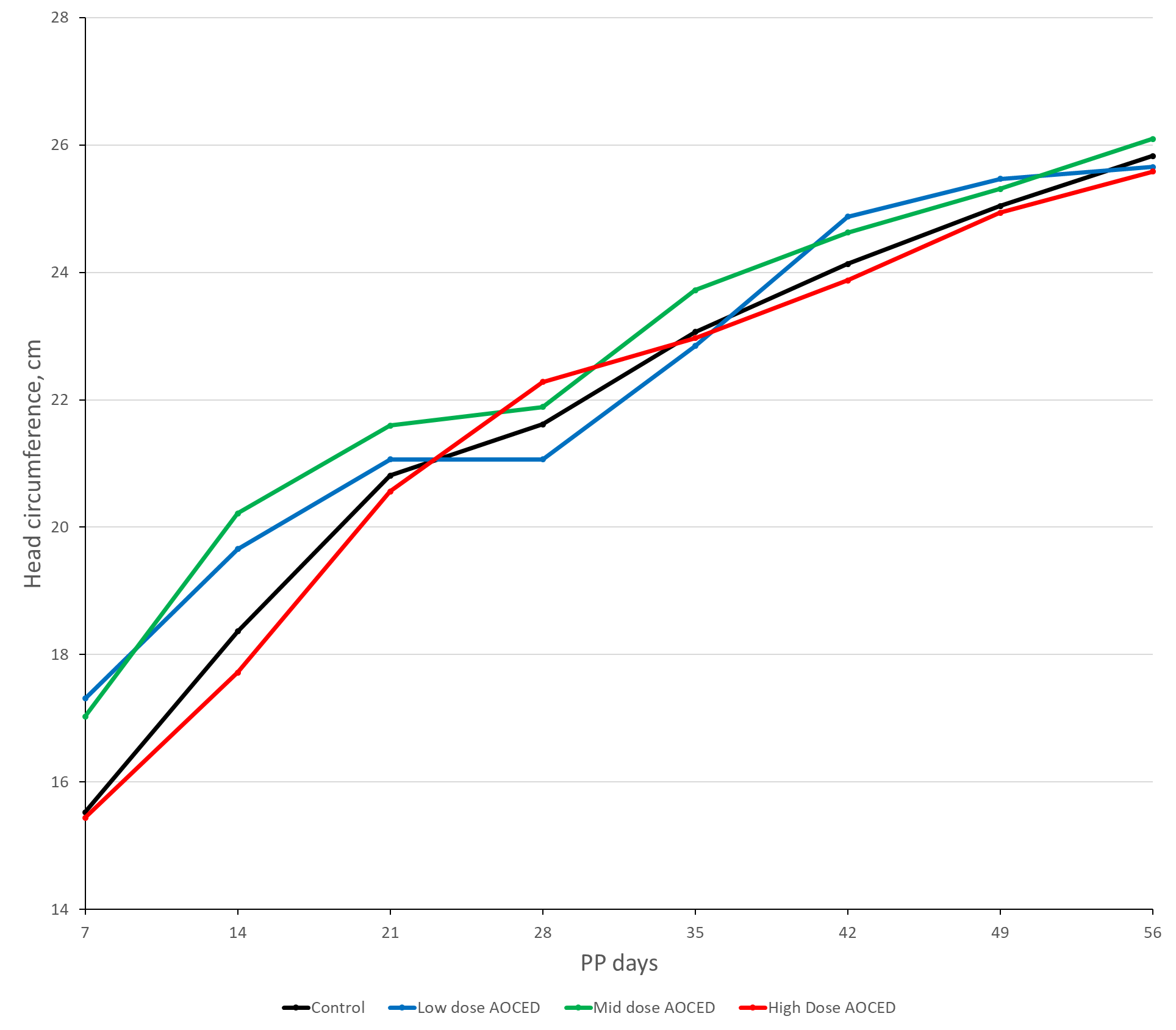

Supplement: S1 Fig — There were no statistically significant differences between males and females, therefore, genders were combined. Values are shown as group means (n = 10). (TIFF) [file pone.0217794.s009.tiff]
