# Supplementary material for: Safety of a novel feed ingredient, Algal Oil containing EPA and DHA, in a gestation-lactation-growth feeding study in Beagle dogs
Source: PLoS One. 2019 Jun 3;14(6):e0217794. doi: 10.1371/journal.pone.0217794 (PMC6546231; doi:10.1371/journal.pone.0217794)
Supplement: S1 Appendix — (DOCX) [file pone.0217794.s010.docx]

S9 Appendix: Statistical analysis

# General information

The statistical analysis approach for this study was designed with reference to Guidance for Industry #226: Target Animal Safety Data Presentation and Statistical Analysis.

Whenever a continuous variable was measured on more than one pup per litter, an average per litter and sex was computed, reported and the statistical analysis was performed using the average per litter and sex.

The individual food consumption was reported and analyzed as weekly average of daily food intake and as weekly average of achieved food intake.

Statistical analysis was independently performed for the three periods: gestation, pre and post weaning.

Transformations of parameters were used for doing the statistical comparisons when their distribution was clearly skewed. Results were then back-transformed and reported in the original unit.

For repeated measures models, a covariance structure was selected (among the first-order autoregressive (AR(1)); the compound symmetry (CS); and the heterogeneous compound symmetry (CSH)) based on the lowest corrected Akaike’s Information Criterion.

# Models used by parameter

## ANOVA/ANCOVA without repeated measures

A one-way analysis of variance (ANOVA) was performed for the following parameters:

- percentage live pups by litter, percentage malformed pups per litter at PPD 0;
- clinical pathology parameters of F_0_ without baseline values at weaning.

A one-way analysis of covariance (ANCOVA) was performed for the following parameters:

- clinical pathology parameters of F_0_ at weaning

A two-way analysis of variance (ANOVA) was performed for the following parameters:

- body weight for F_1_ at PPD 0

## ANOVA/ANCOVA with repeated measures

A two-way repeated measures analysis of variance (ANOVA) was performed for the following parameters:

- body weight for F_0_ during post-partum (lactation phase);
- body weight changes for F_0_ during gestation and post-partum (lactation phase);
- body temperature for F_0_ during post-partum (lactation phase);
- heart rate for F_0_ during post-partum (lactation phase);
- respiratory rate for F_0_ during post-partum (lactation phase);
- food consumption for F_0_ and F_1_ combined (by cage) during post-partum (PPD 0 to weaning);
- food consumption F_1_ during the first 2 weeks post-weaning

A two-way repeated measures analysis of covariance (ANCOVA) was performed for the following parameters:

- post-start of treatment results of body weights F_0_ during gestation
- post-start of treatment results of food consumption F_0_ during gestation
- post-start of treatment results of body temperature F_0_ during gestation;
- post-start of treatment results of heart rate F_0_ during gestation;
- post-start of treatment results of respiratory rate F_0_ during gestation

A three-way repeated measures analysis of variance (ANOVA) was performed for the following parameters:

- body weight and body weight change for F_1_ post-weaning;
- food consumption for F_1_ after the first 2 weeks post-weaning;
- clinical pathology parameters of F_1_;
- body temperature for F_1_ post-weaning;
- heart rate for F_1_ post-weaning;
- respiratory rate for F_1_ post-weaning;
- height and height change for F_1_ post-weaning;
- head circumference and head circumference change for F_1_ post-weaning.

A three-way nested repeated measures analysis of variance (ANOVA) was performed for the following parameters (pups nested within litter) including only the puppies selected at culling:

- body weight and body weight change for F_1_ pre-weaning;
- height and height change for F_1_ pre-weaning;
- head circumference and head circumference change for F_1_ pre-weaning.
